# Beyond Alignment: Static Coronal Alterations Do Not Predict Dynamic Foot Loading or Spatiotemporal Gait Patterns After Unilateral Total Knee Replacement—A Prospective Study

**DOI:** 10.3390/bioengineering13020134

**Published:** 2026-01-23

**Authors:** Dimitrios Ntourantonis, Ilias Iliopoulos, Konstantinos Pantazis, Angelos Kaspiris, Zinon Kokkalis, John Gliatis, Elias Panagiotopoulos

**Affiliations:** 1 Emergency Department, University Hospital of Patras, 26504 Patras, Greece; 2 Department of Medicine, School of Health Sciences, University of Patras, 26504 Patras, Greece; 3 Department of Orthopaedic, Famagusta (Ammochostos) General Hospital, 5284 Paealimni, Cyprus; 4 Department of Orthopaedic, Central Hospital of Karlstad, 65185 Karlstad, Sweden; 5 Third Department of Orthopaedic and Medical School, National Kapodistrian University of Athens, 14561 Athens, Greece; 6 Department of Orthopaedic, University Hospital of Patras, 26504 Patras, Greece

**Keywords:** total knee replacement, total knee arthroplasty, baropodometry, knee osteoarthritis, dynamic plantar pressure, spatiotemporal gait parameters, coronal alignment, femorotibial angle

## Abstract

**Background**: Static coronal alignment is considered a key of lower limb biomechanics after total knee replacement (TKR); however, its relationship with dynamic foot loading patterns and gait characteristics remains unclear. The primary objective of this prospective study was to investigate whether there is a correlation between dynamic plantar pressures and spatiotemporal parameters of gait and the coronal alignment of the lower limb after unilateral TKR for primary knee osteoarthritis (KOA). **Methods**: Thirty-two consecutive patients scheduled for TKR were evaluated preoperatively and at six months postoperatively. Changes in plantar pressure distribution and spatiotemporal gait parameters were collected using a multiplatform plantar pressure analysis system (PPAS), while coronal alignment was assessed using the femorotibial angle (FTA). Relationships with preoperative, postoperative, and correction-related alignment measures were examined using non-parametric statistical methods. **Results**: Dynamic plantar pressures and spatiotemporal gait parameters were not found to be consistently associated with pre- or postoperative values of FTA, respectively. Furthermore, the degree of correction did not appear to influence baropodometric outcomes. **Conclusions**: Static coronal alignment, as defined by the FTA, was not found to be consistently associated with dynamic plantar pressure patterns or spatiotemporal gait parameters at six months following unilateral TKR in our study population. These findings highlight the potential limitations of using solely static radiographic markers to evaluate complex functional outcomes such as gait.

## 1. Introduction

Until recently, there was a belief among orthopedic surgeons that restoration of coronal lower limb alignment after total knee replacement (TKR) to as close to neutral as possible was the key to achieving improved clinical results, along with implant longevity [1,2,3,4,5]. Deviations from neutral alignment have been correlated with lower implant survival rates, suboptimal functional outcomes, and elevated wear rates, especially in earlier polyethylene implant designs [1,2,3,6,7,8,9]. This alignment paradigm was largely supported by biomechanical observations linking static limb alignment in the standing position to dynamic loading patterns during gait, particularly the knee adduction moment [10].

Recent studies, however, have started to challenge this assumption, with growing evidence suggesting that neutral coronal alignment may not be as crucial to functional recovery as previously assumed [11,12,13,14,15,16]. Instead, other factors appear to have a greater influence on postoperative function [13,17].

On the other hand, foot biomechanics have long been considered to be closely related to mechanical alignment of the lower limb. Chandler and Moskal were among the first to suggest that foot posture and loading patterns might be adapted in predictable ways following changes in limb alignment after TKR [18]. It is believed that certain adaptive changes occur in both joints (knee and ankle) as a compensatory response to abnormalities observed on either side [19,20,21]. When there is a deviation in the axis in the frontal plane at the knee joint towards varus or valgus, compensatory adaptations occur in the foot to achieve an optimal load distribution among the hip, knee, and ankle [19,21]. Recent movement biomechanics studies, even outside TKR populations, show that ankle motion and knee joint mechanics are interrelated, such that altered ankle behavior can influence knee loading patterns across dynamic tasks [22]. However, the precise mechanisms by which the foot and subtalar joint adapt to a specific deviation in the knee varus or valgus are not yet fully understood [20,21].

Gait analysis has been used extensively to evaluate the impact of lower limb alignment after TKR and its relationship with gait changes; however, less technically demanding gait evaluation techniques such as baropodometry have not been studied systematically in this field. Baropodometry essentially exploits the application of Newton’s third law, according to which action and reaction are equivalent. As people stand or walk, forces interact between the body and the ground. The evaluation of these forces is the basic principle of foot pressure analysis and can help to understand the loads exerted on the human body during typical activities such as standing or walking, as well as during more intense scenarios such as sports activities [23]. Zhang et al. reported that pressure distribution patterns during the gait cycle can be influenced by changes in knee alignment [24]. Regarding the spatiotemporal parameters of gait, which can also be monitored with the use of plantar pressure analysis systems (PPASs), studies have shown that individuals with more neutral alignment post-TKR tend to exhibit more normalized gait patterns, which are reflected in their spatiotemporal gait metrics [25,26].

Importantly, most existing studies have relied on categorical alignment groupings or descriptive comparisons, while continuous correlation analyses between static coronal alignment measures and dynamic plantar pressure or spatiotemporal gait parameters after TKR remain largely unexplored.

The primary objective of this study was to determine whether there is a correlation between dynamic plantar pressures and spatiotemporal parameters of gait and the coronal alignment of the lower limb after unilateral TKR for primary knee osteoarthritis (KOA). The main hypothesis of the study was that changes in the lower limb axis in the coronal plane, as represented by a static parameter such as the FTA, are not reflected in dynamic foot loading patterns or spatiotemporal gait parameters, as more complex and sophisticated neuromuscular mechanisms are involved during gait which cannot be predicted by a single static parameter.

## 2. Materials and Methods

This study is a part of a prospective, single-center, academic observational protocol that was designed to evaluate plantar pressure distribution and gait parameters in patients undergoing primary unilateral cemented TKR using a posterior cruciate ligament-preserving prosthesis. The broader study included both static and dynamic preoperative baropodometric assessments at a defined postoperative interval [12].

The study protocol was approved by the Ethics Committee of University Hospital of Patras—Greece (IRB number: 265/7-6-23), ensuring compliance with ethical standards and patient safety. All participants provided written informed consent, and all surgical procedures were performed by the same surgeon.

A pre hoc power analysis was performed using G*Power 3.1 [27] to estimate sample size for paired comparisons. To estimate the required size, the Cohen’s dz (0.5), α (0.05), and 1 − β (0.8) values were used. Based on these assumptions, the minimum number of participants was calculated to be 27.

### 2.1. Inclusion and Exclusion Criteria

#### 2.1.1. Inclusion Criteria

(a) Primary, unilateral, grade 3–4 KOA according to the Kellgren–Lawrence Classification (KLC) [28], (b) clinical absence of spinal deformities (kyphosis and/or lordosis) and lower limb deformities (thigh, tibia and foot), (c) ability to walk independently and without the use of a walking aid for at least 20 m before surgery, (d) age > 60 years.

#### 2.1.2. Exclusion Criteria

(a) Pre-existing neurological deficits that could affect posture and/or gait patterns, (b) previous orthopedic surgery on the lower extremities and/or spine, (c) lack of consent, (d) significant complications during surgery and postoperative period, including (i) postoperative infections and (ii) intraoperative and periprosthetic fractures, (e) any other condition during the period of participation in the protocol impacting posture and gait patterns, such as trauma to the lower extremities and/or spine or neurological conditions.

### 2.2. Surgical Intervention

All surgeries were performed by the same senior knee surgeon using a medial parapatellar approach, with tourniquet application and lateral patella retraction. All implants were cemented. The patella was resurfaced when deemed necessary [29,30,31]. All patients followed the standard surgeon-directed rehabilitation program of our institution supervised by the rehabilitation clinic of our hospital. The program included early range-of-motion exercises from the first postoperative day, strengthening exercises, gait training, and partial weight bearing until the third postoperative week. A home-based physiotherapy program twice weekly for an additional four weeks was prescribed afterwards.

### 2.3. Pre- and Post-Operative Patient Evaluation

All participants underwent two evaluations: one immediately preoperatively (pr) and another approximately six months postoperatively (po). The first evaluation aimed to capture the prior radiographic and gait characteristics, as close to the surgical intervention as possible, as these would be affected by the progression of osteoarthritis [18,19,20], while the second evaluation point was chosen to allow sufficient recovery, as most gait changes after TKR occur within the first six months [32,33,34].

#### 2.3.1. Radiological Examination

All participants underwent an anteroposterior (AP) standing, weight-bearing radiograph (SWBR) of the affected knee joint (AKJ) on a large radiological cassette (356 mm × 432 mm) at both timepoints using the same radiographic unit. The results were stored both on film and in DICOM^®^ [35] (Digital Imaging and Communications in Medicine) digital format for further analysis. For radiological grading of osteoarthritis, all participants were classified according to the KLC [28] based on preoperative radiographs assessed by the operating surgeon.

FTA measurements were performed using the free electronic program Roentgen Monographic Analysis (RoMAn, version V1.70) [36,37] and were based on the original DICOM files. Prior to analysis, image calibration was performed using the known physical height of the radiographic cassette (432 mm) to establish a pixel-to-millimeter conversion scale. Calibration was performed before image distribution, and all images were provided to the observers already calibrated by an independent member of the research team not involved in the measurements.

Two independent, blinded senior orthopedic residents (Observers A and B) performed the measurements according to the method of Petersen and Engh [38]. Each observer conducted two independent evaluations of each examination, separated by a 15-day interval. To minimize recall bias, images were anonymized and redistributed using different random file names and randomized order for each measurement session, with cross-distribution of image sets between observers [39,40]. Final preoperative and postoperative FTA values were calculated as the means of both observers’ measurements. Based on these, knees were classified as neutral (N) (172.8–177.6°), varus (VR) (>177.6°), or valgus (VL) (<172.8°) [38,41,42].

#### 2.3.2. Dynamic Baropodometric Analysis

Dynamic baropodometric evaluation was performed using a modular multi-platform plantar pressure analysis system (PPAS) (model: MPS; Loran Engineering Srl, Bologna, Italy) [43], rebranded locally by the distributor (Comex; model MPS, Athens, Greece). The hardware and software specifications are identical to the original MPS system and differ only in local branding. The system comprised four interconnectable platforms, with total dimensions of 2000 mm × 540 mm × 7 mm and 9216 resistive pressure sensors (9 mm × 9 mm each), operating between 50 and 100 Hz.

Measurements were obtained using the mid-gait protocol [44,45], and data were collected for both feet. Average and maximum plantar pressures were recorded, with average values additionally measured for the forefoot (FF), midfoot (MF), and hindfoot (HF) regions. Before baropodometric analysis, all participants were informed about the procedure and asked to remove their shoes and socks [46]. Participants were allowed to familiarize themselves with the procedure through trials, walking over the platform at their own comfortable pace, starting from an adjusted position to allow for at least three to five steps (approximately two meters) before reaching the analysis platform. During the analysis, the platform was left uncovered, and participants were instructed not to look down but to focus on a fixed point ahead to prevent targeting the platform—although this does not seem to affect the results [47]. Baropodometric analysis was performed by an independent member of the team who did not take part in any other part of the study.

Gait cycles were recorded with alternating leading legs across three valid trials, as per European GAITRite^®^ Network guidelines [46]. Each limb was analyzed independently; no within-subject comparisons were performed. PPAS was calibrated using a zero-calibration procedure immediately prior to each measurement session, both preoperatively and postoperatively, in accordance with standard plantar pressure analysis protocols [44,45]. To minimize the potential impact of sensor drift, all recordings were acquired during independently calibrated sessions at each time point. For dynamic analysis, the system operated at the upper limit of the manufacturer-specified sampling rate range (100 Hz).

**Figure 1:**
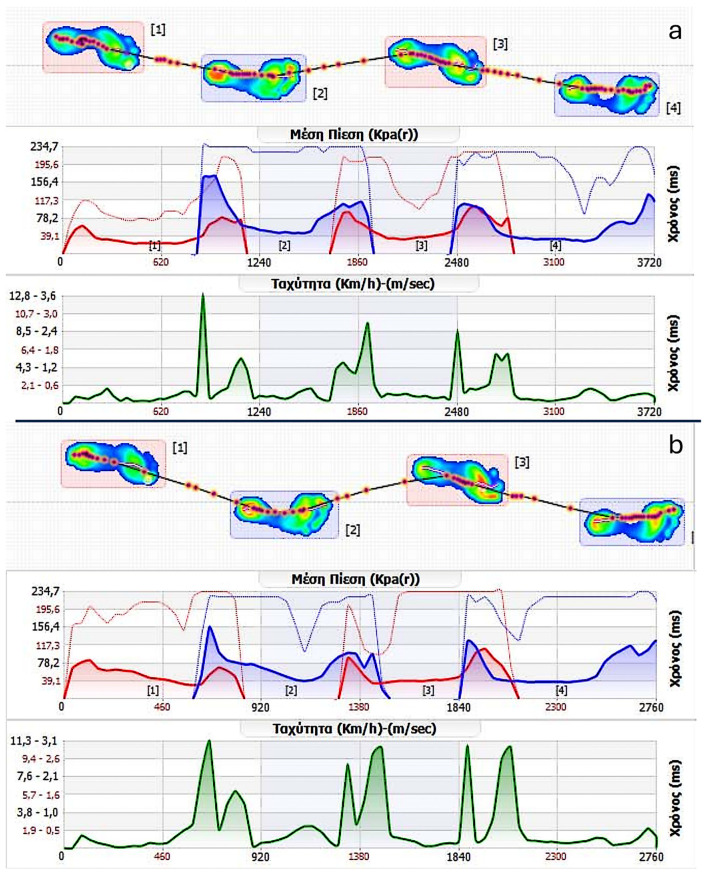
Software-generated output of dynamic plantar pressure analysis during walking before (**a**) and six months after (**b**) unilateral total knee arthroplasty for left knee osteoarthritis. The same analysis protocol and software-generated output were used in both conditions, allowing direct visual comparison of preoperative and postoperative gait characteristics. (Μέση Πίεση: Average Plantar Pressure; Ταχύτητα: Speed; Χρόνος: Time).

Data on baropodometry were collected using “Biomech Studio” Version 1 (Loran Engineering Srl., Bologna, Italy) [43]. The software employs the manufacturer’s validated internal algorithms. Plantar regions (forefoot, midfoot, and hindfoot) were automatically defined by the software. No additional custom filtering, smoothing, or manual post-processing of raw pressure signals was applied (Figure 1). Data were organized in a Microsoft Excel spreadsheet (Microsoft Office 365, version 2310, Redmond, WA, USA) [48] for further processing. All examined baropodometric and spatiotemporal parameters and their definitions can be found in Table 1.

### 2.4. Statistical Analysis

The intra-class correlation coefficient (ICC) was calculated to assess inter- and intra-observer agreement for FTA measurements, using a two-way mixed effects model, while the Shapiro–Wilk test was used to evaluate the normality of all continuous variables. All quantitative variables that showed dependence on somatometric characteristics were normalized and presented as means ± standard deviation (SD), while categorical variables are presented as absolute (n) and relative (%) frequencies.

Paired Student’s *t*-tests and McNemar tests were used for time comparisons. Spearman’s rank correlation coefficients (ρ) were used to explore associations between FTA values (pr, po) and plantar pressures or spatiotemporal parameters of gait at the same time intervals, and between ΔFTA and |ΔFTA| (Δ = po − pr). Benjamini–Hochberg false discovery rate (FDR) control was applied, and adjusted q-values were calculated where necessary. A significance level of q < 0.05 was used to determine statistical significance after correction.

Statistical comparisons of spatiotemporal gait parameters and dynamic plantar pressures across Knee Alignment (KA) groups were performed using the Kruskal–Wallis test, and post hoc pairwise comparisons were conducted using the Dunn–Bonferroni method in cases of significant group differences. For graphical illustration purposes, simple linear regression lines and coefficients of determination (R^2^) were calculated and displayed in scatter plots to visualize potential trends between variables.

Each limb was analyzed independently. Pre- and postoperative comparisons were performed within the same limb (affected and unaffected separately), and no statistical comparisons were made between limbs. All reported *p*-values are two-tailed, with statistical significance set at *p* < 0.05. Statistical analyses were performed using IBM SPSS Statistics [version 28.0.1.1(14)]; IBM Corporation, Armonk, NY, USA) [49].

## 3. Results

A total of 35 patients who met the inclusion criteria were preoperatively included in the study. Over the six-month postoperative follow-up period, a total of three patients were excluded from the final analysis for different reasons (one patient was excluded immediately after TKR due to the use of a posterior-stabilized prosthesis, another sustained an ipsilateral peritrochanteric hip fracture on the fourth postoperative month, and one failed to attend the six-month re-evaluation visit).

Thirty-two patients were included in the final analysis; 68.8% were female (n = 22), with a mean age of 72.8 years (SD = 6.6). The mean body mass index (BMI) was 27.1 kg/m^2^ (SD = 2.0), and 81.3% of participants were classified as overweight. In 17 patients, the left knee was affected, and the majority (n = 23) were graded as stage 4 according to the KLC [34]. Patients were evaluated preoperatively 2.13 days on average before TKR (SD = 1.13) and postoperatively 198.25 (SD = 10.39) days on average after TKR. The full demographic data of our study group is presented in Table 2. The excluded patients had comparable characteristics to those analyzed, with no meaningful differences in age, BMI, or sex distribution.

High intra- and inter-observer reliability was observed for FTA measurements. The lowest intra-observer ICC was 0.852 (observer 2, postoperative), while the highest was 0.890 (observer 1, preoperative). The inter-observer reliability was 0.920 and 0.876 for preoperative and postoperative measurements, respectively, indicating excellent agreement across both evaluations (Table S1).

A mean change of −0.69° ± 4.12° in the preoperative FTA was observed at the six-month follow-up after TKR and did not reach statistical significance. KA of the AKJs was classified as VL in 12 cases, N in five, and VR in 15, according to the preoperative FTA measurements. After TKR, the distribution of KA changed significantly; only two knees were categorized as VL, and five as VR, while the vast majority (n = 25) (*p* = 0.001) were classified as neutral (Table 3). These changes reflected individual alignment shifts toward the neutral range despite the absence of a significant net change in the group mean angle.

**Figure 2:**
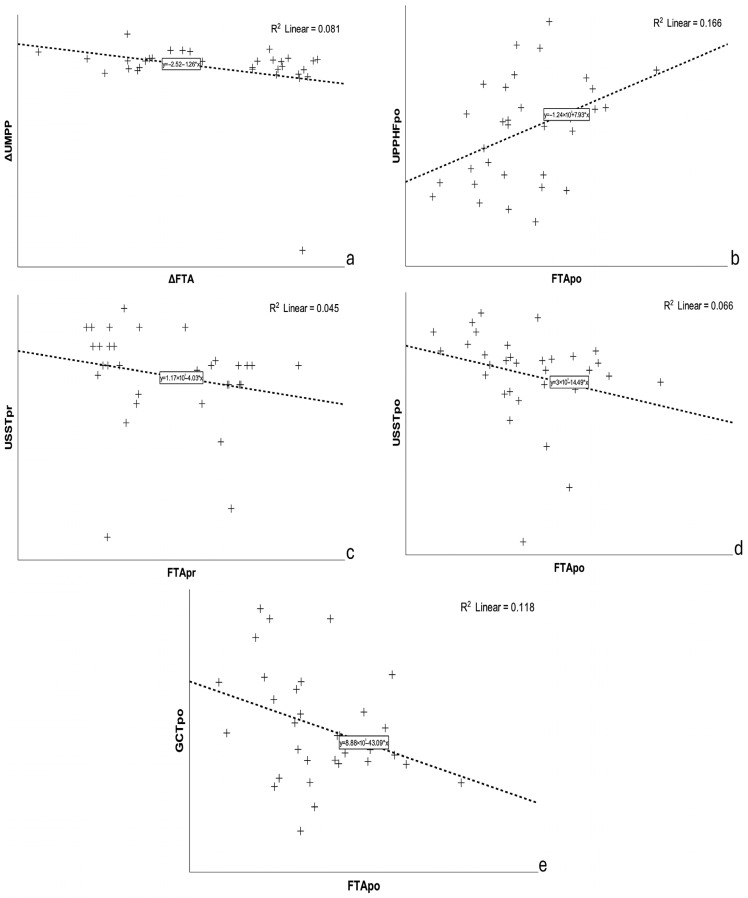
Scatter plots illustrating the relationship between static coronal alignment parameters and selected dynamic plantar pressure and spatiotemporal gait parameters. Linear regression lines and corresponding R^2^ values are shown for each panel (**a**–**e**), demonstrating the absence of meaningful correlations. Δ: postoperative—preoperative values; UMPP: unaffected maximum plantar pressures; UPPHF: unaffected plantar pressure hindfoot; USST: unaffected single support time; GCT: gait cycle time; FTA: femorotibial angle; pr: preoperative; po postoperative; UMPP and UPPHF values in kilopascal; USST and GCT values in millisecond; FTA values in (°).

Spearman’s correlation analysis revealed only one moderate, statistically significant correlation between ΔUMPP and ΔFTA (ρ = −0.365, *p* = 0.040) (Figure 2a). No statistically significant differences were observed between the rest of the dynamic plantar pressure measurements and FTA in both evaluations (all *p*-values > 0.05). As regards dynamic plantar pressures in specific regions of the foot, FTApr showed weak, non-significant correlations (*p* > 0.05) with both affected and unaffected plantar pressures (APP, UPP) across the FF, MF, and HF regions. Similarly, FTApo was not correlated with most plantar pressure parameters, except for the unaffected HF plantar pressure (UPPHFpo), for which there was a weak positive correlation (ρ = 0.394, *p* = 0.026) (Figure 2b). ΔFTA and |ΔFTA| between evaluation intervals did not show significant correlations with any of the changes in plantar pressure parameters (ΔAPP, ΔUPP) across the FF, MF, and HF regions (*p* > 0.05). Expanding our analysis to FTA and spatiotemporal gait parameters, we found moderate, statistically significant correlations for USST (pr/po) with the corresponding FTA values [(ρ = −0.379, *p* = 0.033), (ρ = −0.449, *p* = 0.010)] (Figure 2c,d), and for GCTpo with FTApo (ρ = −0.382, *p* = 0.031) (Figure 2e). However, after correction for multiple comparisons using the Benjamini–Hochberg procedure, none of the above-mentioned correlations remained statistically significant (all q-values > 0.05) (Table S2).

**Figure 3:**
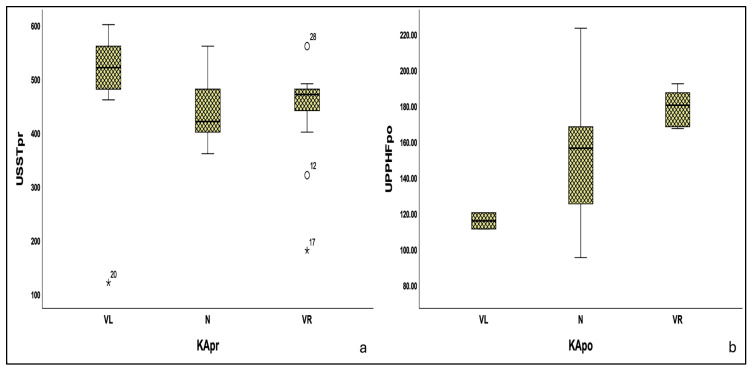
Boxplot distributions of (**a**) preoperative USST and (**b**) postoperative UPPHF values across the corresponding alignment groups. Numbers indicating individual patients USST: unaffected single support time; UPPHF: unaffected plantar pressure hindfoot; KA: knee alignment; pr: preoperative; po: postoperative; VL: valgus; N: neutral; VR: varus; USST values in millisecond; UPPHF values in kiloPascal; o: outlier (values between 1.5 and 3 interquartile ranges); *: extreme outlier (values >3 interquartile ranges).

A Kruskal–Wallis analysis was performed to explore potential differences in dynamic plantar pressures and spatiotemporal gait parameters among different KA groups (VL, N, VR) in both evaluations. The results did not reveal statistically significant differences in the majority of the parameters examined. Only certain variables [USSTpr (*p* = 0.030), UPPHFpo (*p* = 0.039)] showed nominal statistical significance differences across the corresponding KA groups at the respective time points, (Figure 3). Following post hoc pairwise comparisons with Dunn–Bonferroni correction, statistical significance was retained only for USSTpr on the VL-VR subgroup (adjusted *p* = 0.044), while UPPHF on the same subgroup reached to borderline levels (adjusted *p* = 0.050) (Table 4).

## 4. Discussion

Our findings support our main hypothesis that static coronal alignment, as represented by FTA, may not be a reliable predictor of dynamic plantar pressure distribution or spatiotemporal gait parameters following unilateral TKR.

The small and non-significant mean change in FTA should be interpreted in the context of bidirectional individual alignment corrections: knees with preoperative varus required a reduction in FTA to reach the neutral range, whereas knees with preoperative valgus required an increase, resulting in offsetting changes at the group mean level rather than limited surgical effect.

Although certain correlations between alignment-related variables and plantar pressures or spatiotemporal gait parameters were observed before correction for multiple comparisons, these associations did not remain significant for the majority of the correlated variables after FDR adjustment and should therefore be regarded as exploratory rather than robust relationships. Only isolated findings remained significant after FDR correction and were therefore considered, including differences in USST between the VR and VL subgroups preoperatively (adjusted *p* = 0.044) and UPPHF postoperatively (adjusted *p* = 0.052) within the same alignment subgroup. The overall results indicate the absence of robust and consistent associations among static alignment, dynamic plantar pressure patterns, and spatiotemporal gait parameters. However, the interpretation of these near-significant findings should therefore be made with caution, constrained by the very small number of valgus knees in the postoperative cohort, which limits meaningful alignment-based subgroup comparisons.

This finding aligns with a growing body of literature questioning the previously accepted paradigm that achieving neutral mechanical alignment after TKR guarantees optimal functional results. While early research emphasized the mechanical axis as a key determinant of implant longevity and gait restoration [4,5,50], more recent studies suggest that the relationship between static alignment and dynamic performance is far less straightforward and still debatable [13,51,52,53,54]. Hunt et al., in their study on patients with knee osteoarthritis, concluded that, although the mechanical axis angle of the lower limb provides an accurate static measurement, it does not adequately reflect the dynamic changes that occur during movement and weight-bearing activities [55]. Similarly, Larose et al. highlighted the lack of correlation between static and dynamic alignment, a finding that suggests that static radiographic coronal alignment of the knee does not accurately predict its dynamic behavior [56]. The low prediction value of the dynamic limb behavior has also been reported by Riviere et al. based on 3D motion analyses pre and post TKR of the Hip-Knee-Ankle (HKA) angle [57].

On the other hand, only a few studies have used PPASs to examine the relationship between coronal alignment and the dynamic plantar pressure distribution before and after TKR. Kamenaga et al. compared patients who were kinematically aligned with those who underwent mechanical alignment and found a more even pressure distribution between the medial and lateral rearfoot in kinematically aligned knees; however, they did not explore the correlation of coronal alignment with their results [58]. Another study reported that several dynamic plantar pressure parameters showed a statistically significant decrease in the forefoot and midfoot regions, along with a significant increase in the hindfoot, in neutral and valgus knees postoperatively, but no data was provided regarding the correlation with the degree of correction in these participants [59]. To our knowledge, no study was found testing continuous correlations between plantar pressures or spatiotemporal gait parameters and FTA.

The lack of strong statistically significant correlations in our analysis among FTA, dynamic plantar pressures, and spatiotemporal gait parameters, may be an indication that dynamic gait adaptations involve complex compensatory mechanisms, likely influenced by neuromuscular control, proprioception, and individual preoperative adaptations, rather than by alignment alone. This aligns with the concept that functional adaptation after TKR is primarily neuromuscular rather than geometric [60]. The observation that moderate correlations between ΔUMPP and ΔFTA, FTApr/po and USSTpr/po, FTApo and GCTpo FTA failed to maintain significance after FDR correction underscores the potential for spurious associations when multiple parameters are explored simultaneously.

According to Febrer-Nafría and colleagues, little is known about the relationship and more specifically the relative impact of muscle recruitment strategies vs. implant alignment scenarios on post-TKR joint contact mechanics. Furthermore, knee kinematics seems primarily to be affected by the implant position, but knee contact loading patterns are mainly correlated with variations in muscle activation strategies [60].

On the same content, one of the reasons that TKRs fail to restore function is due to the loss of skeletal muscle to adapt early after TKR [61]. These functional alterations are not completely understood but are hypothesized to be due to reduced neuromuscular activation and skeletal muscle adaptations to KOA for longer periods, such as atrophy and weakness [62,63]. Although neural activation deficits seem to resolve relatively early on the post TKR period, deficits and maladaptations of the skeletal muscles are thought to have a greater impact on long-term functional disability and to persist over longer periods [64] potentially influencing daily activities and gait patterns, despite joint reconstruction and the alignment restoration.

The inclusion of patients with varying radiographic disease severity is an important consideration when interpreting these findings. Although it is believed that KOA progression affects gait patterns, the relationship between radiographic severity, as classified by the KLC, and functional gait behavior remains controversial. Studies reporting weak or inconsistent associations [65,66,67,68,69] while others suggest a potential influence of radiographic disease severity on function and gait [70,71,72]. In patients already fulfilling clinical indications for TKR, factors such as pain, and neuromuscular adaptations [69,73] or even gender-related differences [74] may exert a more prominent influence on dynamic plantar pressure distribution and spatiotemporal gait characteristics than radiographic grade alone.

Different coronal alignment patterns reflect routine clinical practice, as KOA and TKR populations typically present with a wide spectrum of VR, N, and VL alignment, and should be considered when interpreting these findings. Previous biomechanical and clinical studies examining alignment-related outcomes in ΚOA and ΤΚR have consistently included patients across the spectrum of VR, N, and VL alignment, reflecting the real-world distribution of coronal alignment rather than homogeneous subgroups. Within this clinically representative context, the absence of robust or consistent subgroup differences in the present study suggests that coronal alignment category alone may not be a dominant predictor of postoperative dynamic plantar pressures and spatiotemporal gait parameters.

In addition, the inclusion of patients with and without patellar resurfacing, also reflects routine clinical practice and was not expected to substantially influence gait-related outcomes during level walking according to the literature [29,30,31].

Our methodology provides a practically superior, clinically feasible and cost-effective method to evaluate gait dynamics alternative to complex gait laboratory protocols. We have made a consistent effort to keep our protocol simple and to combine all the data that could be extracted from the PPAS and to correlate them with FTA, both pre- and postoperatively. We deliberately avoided including pressures from specific subregions of the foot in our analysis (metatarsal heads, toes, lateral and medial hindfoot), as we strongly believed that this would unnecessarily increase the complexity of the study. Furthermore, previous studies have not succeeded in correlating plantar pressure changes after TKR with the degree of correction, even in static baropodometric analysis [12].

The absence of robust correlation even with a simple, functionally oriented tool such as a PPAS supports its potential clinical value not as a surrogate for alignment, but as a modality capturing functionally expressed plantar loading and gait patterns. Even though baropodometry is considered an objective measurement method, its results are complex and require specialized knowledge for accurate interpretation [12]. In other words, baropodometry should not be viewed as a tool designed to directly quantify coronal alignment outcomes, but rather as a method for assessing functional performance that may or may not be influenced by static anatomical alignment.

Our study had several notable strengths. The prospective character, which included patients who underwent unilateral TKR using the same CR implant; the use of a validated PPAS; and the comprehensive analysis of dynamic plantar pressure and spatiotemporal gait parameters acquired under a strict mid-gait protocol, taken together, the standardized data processing workflow and the careful handling of paired measurements [75], strengthen the reliability of our findings.

To our knowledge, this is the first prospective study to test the continuous correlation between FTA changes after unilateral TKR and dynamic plantar pressures and spatiotemporal gait parameters, collected with the use of a PPAS. This approach provides a functional perspective on how lower-limb alignment interacts with gait adaptation, emphasizing that static radiographic parameters alone cannot predict postoperative functional performance.

However, our study, like any study, had certain limitations. Our major limitation is the moderate sample size, which limited the ability to detect small-to-moderate correlations; therefore, weaker associations cannot be excluded. Accordingly, the findings should be interpreted with caution, and larger studies are needed to further explore these relationships.

The absence of additional dynamic metrics such as knee adduction moments or electromyographic data, which could have provided deeper insight into the neuromechanical pathways, might be considered another limitation of our study, but in reality, reflects a deliberate scope. The present study was designed to maintain a focused and clinically relevant approach, rather than to perform an extensive neuromechanical gait analysis.

Finally, the assessment of coronal alignment was based on the FTA measured from SKWBR can also be considered as a limitation. Although this method has been validated and demonstrates a strong correlation with HKA measured on full-length lower-limb radiographs (r ≈ 0.86 according to Shang et al. [76]), it does not perfectly reflect the true HKA angle, but in can be used as an acceptable alternative to coronal alignment in both clinical and research settings, especially when full-length radiographs are not feasible [41,77]. In addition, the absence of severe coronal deformities (>20° of varus/valgus [78,79]), limited subgroup-level analyses and may restrict extrapolation to cases of extreme malalignment.

## 5. Conclusions

In conclusion, our data indicate that static coronal alignment, as defined by the FTA, was not associated with moderate or strong correlations with dynamic plantar pressure patterns or spatiotemporal gait parameters six months after unilateral TKR in our study population. Considering the study limitations, these findings highlight that static radiographic alignment alone may have limited value in capturing complex functional outcomes such as human gait, and underscore the need for a more comprehensive, function-oriented approach in the assessment of TKR performance. Dynamic alignment tools and neuromuscular assessments should be integrated in future research to better understand the determinants of postoperative gait restoration.

## Figures and Tables

**Table 1:** Definition of abbreviations and measurement units used in gait and plantar pressure analysis.

Abbreviation	Definition		Units
AST	Affected step time	Time interval from toe-off of the affected limb to its subsequent initial contact with the ground.	ms
UST	Unaffected step time	Time interval from toe-off of the unaffected limb to its subsequent initial contact with the ground.	ms
MST	Mean step time	AST/UST average	ms
ASST	Affected single support time	Duration of single-limb support on the affected side	ms
USST	Unaffected single support time	Duration of single-limb support on the unaffected side	ms
MSST	Mean single support time	ASST/USST average	ms
DST	Double support time	Time period during which both feet are simultaneously in contact with the ground	ms
GCT	Gait cycle time	Time required to complete one full gait cycle.	ms
APP	Affected plantar pressure	Average plantar pressure recorded under the affected foot during gait cycle.	kPa
UPP	Unaffected plantar pressure	Average plantar pressure recorded under the unaffected foot during gait cycle.	kPa
AMPP	Affected maximum plantar pressure	Maximum plantar pressure recorded under the affected foot during gait cycle.	kPa
UMPP	Unaffected maximum plantar pressure	Maximum plantar pressure recorded under the unaffected foot during gait cycle.	kPa
Sp	Walking speed	Distance covered per time unit of time during walking.	m/s
ASL	Affected step length	Linear distance between successive initial contacts of the affected foot and the contralateral foot during walking.	cm
USL	Unaffected step length	Linear distance between successive initial contacts of the unaffected foot and the contralateral foot during walking.	cm

ms: millisecond (10^−3^ s); kPa: kilopascal (10^3^ Pascal); m/s: meters per second; cm: centimeter (10^−2^ m).

**Table 2:** Demographic and clinical characteristics of the study participants.

		n	%
Sex			
	Females	22	62.9
	Males	13	37.1
Age (years), mean (SD)		72.7 (6.4)	
Height (cm), mean (SD)		167.5 (7.7)	
Weight (kg), mean (SD)		76.6 (9.5)	
BMI (kg/m^2^), mean (SD)		27.2 (2)	
BMI			
	Normal	4	11.4
	Overweight	28	80.0
	Obese	3	8.6
AKJ			
	Right	15	46.9
	Left	17	53.1
KLC			
	Moderate (3)	11	31.4
	Severe (4)	24	68.6

SD: Standard deviation; cm: centimeter (10^−2^ m); kg: kilogram; BMI: Body Mass Index; AKJ: Affected Knee Joint; KLC: Kellgren-Lawrence Classification.

**Table 3:** Pre- and postoperative comparison of femorotibial angle (FTA) and knee alignment (KA) categories.

	pr	po	*p*
FTA (°), mean (SD)	176.26 (5.37)	175.57 (1.73)	0.305 ^+^
KA, n (%)			
VL	12 (37.5)	2 (6.3)	0.001 ^++^
N	5 (15.6)	25 (78.1)	0.001 ^++^
VR	15 (46.9)	5 (15.6)	0.001 ^++^

^+^ Paired *t*-test; ^++^ McNemar test; *p* significant at the 0.05 level; pr: pre-operative; po: post-operative; ΚA: knee alignment; VL: valgus; N: neutral; VR: varus.

**Table 4:** Post hoc pairwise comparisons of USSTpr and UPPHFpo across coronal knee alignment groups (VL, N, VR) using Dunn’s test with Bonferroni correction.

USSTpr-KApr			UPPHF-KApo	
	*p*	Adj. P ^a^	*p*	Adj. P ^a^
N-VR	0.878	1.000	0.159	0.478
N-VL	0.055	0.164	0.017	0.052
VR-VL	0.015	0.044	0.050	0.150

*p* significant at the 0.05 level ^a^ Significance values have been adjusted by the Bonferroni correction for multiple tests. pr: preoperative; po: postoperative; VL: valgus; N: neutral; VR: varus; USST: unaffected single support time; UPPHF: unaffected plantar pressure hindfoot; KA: knee alignment.

## Data Availability

The dataset presented and analyzed in this study has been uploaded to FigShare.com. DOI will be available on request to the corresponding author.

## Supplementary Materials

The following supporting information can be downloaded at: https://www.mdpi.com/article/10.3390/bioengineering13020134/s1, Table S1: Summary of demographic characteristics, radiographic measurements, and coronal alignment type of the affected lower limb in the study participants, preoperatively and postoperatively. Table S2: Spearman correlation analysis between femorotibial angle (FTA) and plantar pressure as well as spatiotemporal gait parameters, preoperatively (FTApr) and postoperatively (FTApo).

## Abbreviations

The following abbreviations are used in this manuscript in alphabetic order:

AKJ	Affected Knee Joint
AMPP	Affected Maximum Plantar Pressure
AP	AnteroPosterior
APP	Affected Plantar Pressure
ASL	Affected StepLength
ASST	Affected Single Support Time
AST	Affected Step Time
BMI	Body Mass Index
DICOM	Digital Imaging and Communications in Medicine
FF	ForeFoot
FTA	FemoroTibial Angle
GCT	Gait Cycle Time
HF	HindFoot
ICC	Intra-class Correlation Coefficient
KA	Knee Alignment
KLC	Kellgren-Laurence Classification
KOA	Knee OsteoArthritis
MF	MidFoot
MSST	Mean Single Support Time
MST	Mean Step Time
N	Neutral
po	Postoperative
PPAS	Plantar Pressure Analysis System
pr	Preoperative
RoMAN	Roentgen Monographic Analysis
SD	Standard Deviation
Sp	Walking Speed
SWBR	Standing, weight-bearing radiograph
TKR	Total Knee Replacement
UMPP	Unaffected Maximum Plantar Pressure
UPP	Unaffected Plantar Pressure
USL	Unaffected Step Length
USST	Unaffected Single Support Time
UST	Unaffected Step Time
VL	Valgus
VR	Varus
