# Supplementary material for: Beyond Alignment: Static Coronal Alterations Do Not Predict Dynamic Foot Loading or Spatiotemporal Gait Patterns After Unilateral Total Knee Replacement—A Prospective Study
Source: Bioengineering (Basel). 2026 Jan 23;13(2):134. doi: 10.3390/bioengineering13020134 (PMC12938825; doi:10.3390/bioengineering13020134)
Supplement: Supplementary file 1 [file bioengineering-13-00134-s001.zip › bioengineering-4080738 - Table S2.pdf]

| | | Preoperative values vs. FTApr | Postoperative values vs. FTApo |
|---|---|---|---|
| UPP | (ρ) | -0.103 | -0.001 |
| | p | 0.575 | 0.996 |
| | q | 0.897 | 0.996 |
| UPPFF | (ρ) | 0.168 | 0.061 |
| | p | 0.359 | 0.741 |
| | q | 0.756 | 0.864 |
| UPPMF | (ρ) | 0.127 | 0.107 |
| | p | 0.488 | 0.558 |
| | q | 0.864 | 0.864 |
| UPPHF | (ρ) | 0.132 | 0.394 |
| | p | 0.473 | 0.026* |
| | q | 0.864 | 0.485 |
| APP | (ρ) | -0.037 | 0.121 |
| | p | 0.841 | 0.509 |
| | q | 0.897 | 0.864 |
| APPFF | (ρ) | -0.023 | -0.155 |
| | p | 0.899 | 0.396 |
| | q | 0.925 | 0.803 |
| APPMF | (ρ) | -0.035 | -0.123 |
| | p | 0.847 | 0.504 |
| | q | 0.897 | 0.864 |
| APPHF | (ρ) | 0.150 | 0.270 |
| | p | 0.411 | 0.136 |
| | q | 0.803 | 0.688 |
| AMPP | (ρ) | -0.175 | 0.036 |
| | p | 0.338 | 0.845 |
| | q | 0.864 | 0.897 |
| UMPP | (ρ) | -0.083 | 0.264 |
| | p | 0.653 | 0.144 |
| | q | 0.897 | 0.749 |
| AST | (ρ) | -0.050 | -0.244 |
| | p | 0.784 | 0.178 |
| | q | 0.897 | 0.756 |
| UST | (ρ) | -0.127 | -0.350 |
| | p | 0.488 | 0.050 |
| | q | 0.864 | 0.485 |
| ASST | (ρ) | -0.054 | -0.281 |
| | p | 0.768 | 0.119 |
| | q | 0.897 | 0.688 |
| USST | (ρ) | -0.379 | -0.449 |
| | p | 0.033* | 0.010* |
| | q | 0.485 | 0.485 |
| DST | (ρ) | 0.026 | -0.093 |
| | p | 0.889 | 0.614 |
| | q | 0.575 | 0.729 |
| GCT | (ρ) | -0.130 | -0.382 |
| | p | 0.479 | 0.031 |
| | q | 0.864 | 0.485 |
| Sp | (ρ) | 0.081 | 0.341 |
| | p | 0.659 | 0.056 |
| | q | 0.897 | 0.485 |
| ASL | (ρ) | 0.084 | 0.215 |
| | p | 0.647 | 0.237 |
| | q | 0.897 | 0.803 |
| USL | (ρ) | 0.251 | 0.185 |
| | p | 0.166 | 0.312 |
| | q | 0.756 | 0.864 |

ρ denotes Spearman's rank correlation coefficient.
Statistical significance was set at $p < 0.05$.
*q* values correspond to false discovery rate–adjusted *p* values (Benjamini–Hochberg procedure).
FTA: femorotibial angle; pr: preoperative; po: postoperative; UPP: unaffected plantar pressure; FF: forefoot; MF: midfoot; HF: hindfoot; APP: affected plantar pressure; AMPP: affected mean plantar pressure; UMPP: unaffected plantar pressure; AST: affected step time; UST: unaffected step time; ASST: affected single support time; USST: unaffected single support time; DST: double support time; GCT: gait cycle time; Sp: speed; ASL: affected step length; USL: unaffected step length
