# Supplementary material for: Beyond Alignment: Static Coronal Alterations Do Not Predict Dynamic Foot Loading or Spatiotemporal Gait Patterns After Unilateral Total Knee Replacement—A Prospective Study
Source: Bioengineering (Basel). 2026 Jan 23;13(2):134. doi: 10.3390/bioengineering13020134 (PMC12938825; doi:10.3390/bioengineering13020134)
Supplement: Supplementary file 1 [file bioengineering-13-00134-s001.zip › bioengineering-4080738 - Table S1.pdf]

PT	GEN	AGE	AKJ	KLC	FTA PR	KA PR	FTA PO	KA PO	DIF
**1**	F	64	L	4	182.45	VR	177.20	N	-5.25
**2**	F	71	L	4	170.40	VL	174.15	N	3.75
**3**	M	81	L	4	171.78	VL	174.13	N	2.35
**4**	F	83	R	4	181.40	VR	178.35	VR	-3.05
**5**	M	78	R	4	170.55	VL	175	N	4.45
**6**	F	76	R	3	172.80	N	174.98	N	2.18
**7**	F	68	L	4	181.53	VR	180.10	VR	-1.43
**8**	F	81	R	3	172.63	VL	174	N	1.38
**9**	M	74	L	3	178.83	VR	176.40	N	-2.43
**10**	F	72	L	4	187.45	VR	177.68	VR	-9.78
**11**	M	79	L	4	179.25	VR	175.28	N	-3.98
**12**	M	73	R	4	180.85	VR	176.23	N	-4.63
**13**	F	72	L	4	182.55	VR	176.15	N	-6.40
**14**	F	65	R	4	170.83	VL	174.30	N	3.48
**15**	F	80	R	4	180.40	VR	176.08	N	-4.33
**16**	F	63	R	4	180.03	VR	177.98	VR	-2.05
**17**	M	78	R	4	181.73	VR	177	N	-4.73
**18**	F	67	R	3	172.25	VL	174.85	N	2.60
**19**	M	79	L	4	183.08	VR	177.90	VR	-5.18
**20**	M	85	R	4	171.20	VL	175.43	N	4.23
**21**	F	64	R	4	183.50	VR	176.20	N	-7.30
**22**	F	70	R	4	173.85	N	174.98	N	1.13
**23**	M	70	L	3	173.93	N	175.93	N	2
**24**	F	73	L	3	171.38	VL	174.90	N	3.53
**25**	F	76	R	3	171.25	N	175.20	N	3.95
**26**	F	82	L	4	169.88	VL	173.55	N	3.68
**27**	F	65	L	4	169.98	VL	172.63	V	2.65
**28**	F	67	L	3	177.83	VR	173.70	N	-4.13
**29**	F	74	L	4	182.35	VR	177.13	N	-5.23
**30**	M	75	L	3	173.68	N	174.80	N	1.13
**31**	F	63	L	4	169.43	VL	172.38	V	2.95
**32**	M	63	R	3	171.40	VR	173.83	N	2.43
				AVG	176.26		175.57		-0.69
				SD	5.37		1.73		4.12
				RANGE	169.43 - 187.45		172.38 - 180.1		-9.78 - 4.45

PT: patient; GEN: gender; AKJ: affected knee joint; KLC: Kellgren-Lawrence classification; FTA: femorotibial angle; PR: preoperative; KA; knee alignment; PO: postoperative; DIF: difference
